# Melatonin improves maternal sleep deprivation‐induced learning and memory impairment, inflammation, and synaptic dysfunction in murine male adult offspring

**DOI:** 10.1002/brb3.3515

**Published:** 2024-05-03

**Authors:** Yue‐Ming Zhang, Ru‐Meng Wei, Zong‐Yin Li, Xue‐Yan Li, Kai‐Xuan Zhang, Yi‐Jun Ge, Xiao‐Yi Kong, Xue‐Chun Liu, Gui‐Hai Chen

**Affiliations:** ^1^ Department of Neurology (Sleep Disorders) The Affiliated Chaohu Hospital of Anhui Medical University Hefei Anhui China; ^2^ Department of Neurology The Second People's Hospital of Hefei Hefei Hospital Affiliated to Anhui Medical University Hefei Anhui China

**Keywords:** cognition, inflammation, maternal sleep deprivation, synaptic proteins

## Abstract

**Introduction:**

Maternal sleep deprivation (MSD), which induces inflammation and synaptic dysfunction in the hippocampus, has been associated with learning and memory impairment in offspring. Melatonin (Mel) has been shown to have anti‐inflammatory, antioxidant, and neuroprotective function. However, the beneficial effect of Mel on MSD‐induced cognitive impairment and its mechanisms are unknown.

**Methods:**

In the present study, adult offspring suffered from MSD were injected with Mel (20 mg/kg) once a day during postnatal days 61–88. The cognitive function was evaluated by the Morris water maze test. Levels of proinflammatory cytokines were examined by enzyme‐linked immunosorbent assay. The mRNA and protein levels of synaptic plasticity associated proteins were examined using reverse transcription‐polymerase chain reaction and western blotting.

**Results:**

The results showed that MSD impaired learning and memory in the offspring mice. MSD increased the levels of interleukin (IL)‐1creIL‐6, and tumor necrosis factor‐α and decreased the expression levels of brain‐derived neurotrophic factor, tyrosine kinase receptor B, postsynaptic density protein‐95, and synaptophysin in the hippocampus. Furthermore, Mel attenuated cognitive impairment and restored markers of inflammation and synaptic plasticity to control levels.

**Conclusions:**

These findings indicated that Mel could ameliorate learning and memory impairment induced by MSD, and these beneficial effects were related to improvement in inflammation and synaptic dysfunction.

## 1 INTRODUCTION

Sleep is a universal phenomenon characterized by reduced movement and responsiveness, from which an individual could be awoke by stimulation. Sufficient sleep is beneficial to the restoration of physiological homeostasis, whereas sleep dysfunction affects behavior, cognition, immune function, hormone fluctuation, and metabolic processes (Brown, 2012; Hu et al., 2003; Vargas & Lopez‐Duran, 2017). With societal development, people's sleep patterns are disturbed by work load, frequent shift work, and the excessive use of electronic devices, including mobile phones and computers (Aldabal & Bahammam, 2011; Calamaro et al., 2009; Xie et al., 2020). Furthermore, during pregnancy, the sleep patterns change owing to pregnancy‐associated anatomical, physiological, and hormonal alterations (Kadam et al., 2023). These changes, combined with work load, increase the risk of sleep dysfunction in pregnant women, which could lead to several adverse consequences in their offspring.

Previous studies have shown that maternal sleep restriction can reduce cardiac baroreflex response, cause hypertension, and alter renal function in rat offspring (Lima et al., 2014). Pups who suffered from maternal rapid eye movement sleep deprivation during late pregnancy showed an increased percentage of active sleep and decreased percentage of quiet sleep and wakefulness during postnatal days (PND) 1–10 (Aswathy et al., 2018). Maternal sleep deprivation (MSD) is also known to induce anxiety, depression, cognition, and altered sexual behavior in offspring (Alvarenga et al., 2013; Peng et al., 2016).

Despite MSD having been shown to change biochemistry and behavior in offspring, the molecular biological mechanisms underlying these effects remain unknown. One study demonstrated that MSD significantly activates hippocampal microglia with the subsequent overproduction of proinflammatory cytokines including interleukin (IL)‐1β, IL‐6, and tumor necrosis factor‐α (TNF‐α), which could lead to neurogenesis impairment and learning and memory dysfunction (Zhao et al., 2014). Moreover, anti‐inflammatory therapy is known to attenuate microglial pro‐inflammatory activation and improve cognitive dysfunction induced by MSD in offspring (Zhao et al., 2015). Increasing evidence indicates that hippocampal synaptic plasticity plays an essential role in modulating learning and memory. An association between the MSD‐induced spatial learning and memory decline and impaired hippocampal CA1 long‐term potentiation has also been reported (Peng et al., 2016). Furthermore, synaptic plasticity‐associated proteins, such as brain‐derived neurotrophic factor (BDNF), postsynaptic density protein‐95 (PSD‐95), and synaptophysin (SYN), have been found to be involved in hippocampal synaptic plasticity, synaptic maturation, and synaptic transmission (de Oliveira et al., 2023). Our previous studies report that MSD decreases the expression levels of BDNF, PSD‐95, and SYN in the hippocampus, accompanied by poor performance in the Morris water maze test (Zhang, Cheng, et al., 2022; Zhang, Wei, Ni, et al., 2023; Zhang, Wei, Li, et al., 2023). Given the above evidence, therapeutic methods focusing on reducing the inflammatory response and improving synaptic plasticity may effectively ameliorate cognitive impairment induced by MSD.

Melatonin (Mel) is a neurohormone secreted by the pineal gland and released into the cerebrospinal fluid and circulation mainly at night (Ruddick et al., 2006). Mel was initially reported to control circadian rhythms and seasonal breeding in mammals. However, recent studies show that Mel has anti‐inflammatory, antioxidant, anti‐apoptosis, and cognitive improvement effects in different pathological models owing to its ability to easily cross the blood–brain barrier (He et al., 2010; Olayaki et al., 2018; Reiter et al., 2016). Mel effectively ameliorates lead‐induced anxiety and depression‐like behaviors and memory acquisition impairment by improving oxidative stress and neuroinflammation in the hippocampus (Omeiza et al., 2021). Mel also prevents the downregulated expression levels of *N*‐methyl‐d‐aspartate receptor subunit (NR2A/B), BDNF, calcium/calmodulin‐dependent protein kinase II, and SYN and attenuates cognitive impairment caused by chronic administration of dexamethasone (Tongjaroenbuangam et al., 2013). Moreover, Mel is beneficial for improving neuronal growth, neuroplasticity, and neurogenesis through upregulating the protein expression levels of PSD‐95 and growth‐associated protein 43 (Juan et al., 2014). However, the role of Mel on MSD‐induced learning and memory impairment and its associated mechanisms are unclear.

In the present study, we hypothesize that Mel improves MSD‐induced cognitive dysfunction by modulating inflammation and synaptic function in the hippocampus. Therefore, offspring mice who had been subjected to MSD during gestational days (GD) 15–21 were administered Mel during PND 61–88. Their cognitive function was then evaluated by the Morris water maze test. Finally, markers of inflammation (IL‐1β, IL‐6, and TNF‐α) and synaptic function (BDNF, TrkB, PSD‐95, and SYN) were examined in the hippocampus.

## 2 MATERIALS AND METHODS

### 2.1 Animals

**FIGURE 1:**
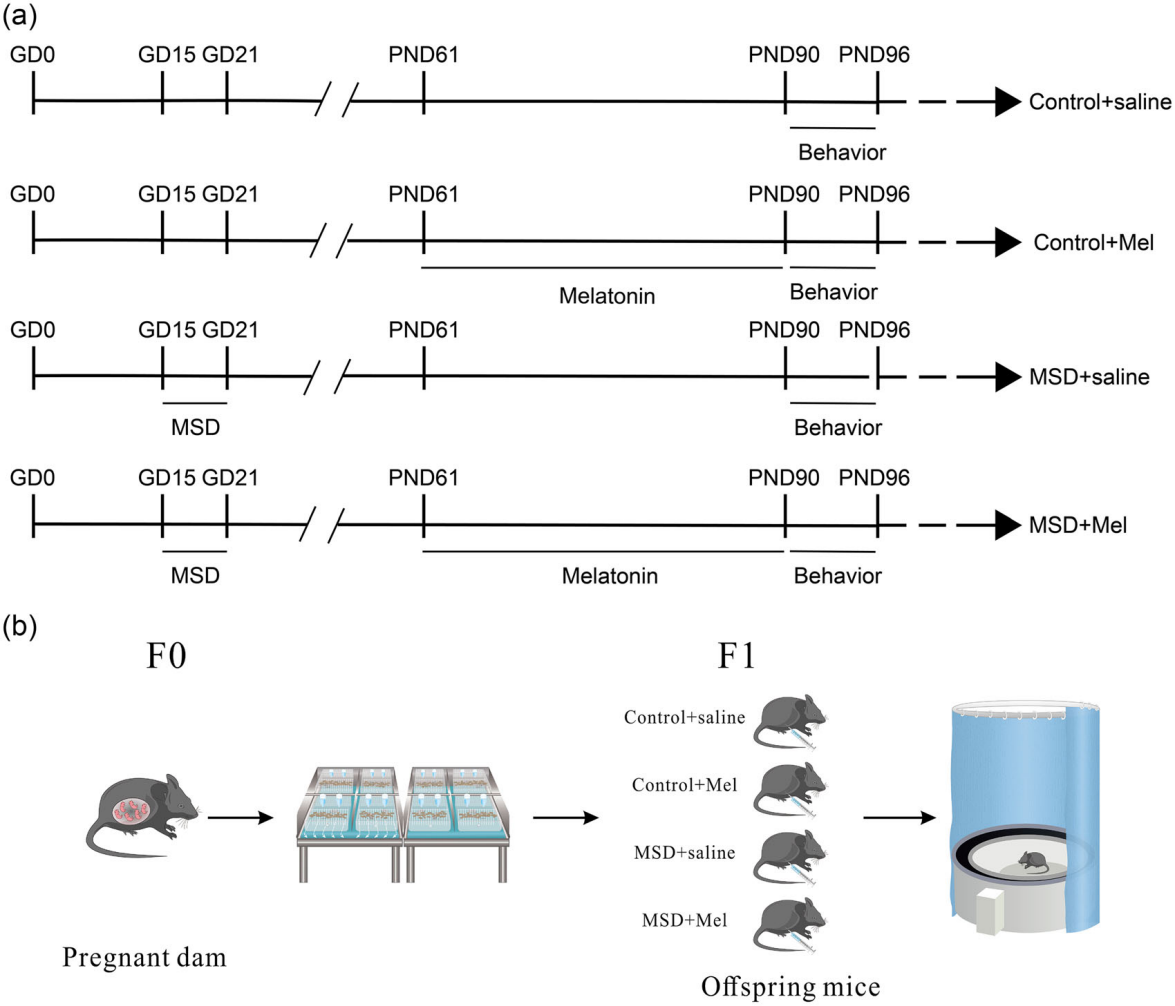
The experimental protocol: (a) the timeline of experimental events; (b) schema for the maternal sleep deprivation machine in laboratory conditions. GD, gestational day; Mel, melatonin; MSD, maternal sleep deprivation; PND, postnatal day.

All c57bl/6 mice were obtained from the Beijing Vital River Laboratory Animal Company. After 1 week of acclimatization, male and female mice were mated (male: female = 1:2). The vagina of each female mouse was examined the following morning; the day when a vaginal plug was observed was recorded as day 0 of gestation (GD 0). Pregnant mice were housed individually and underwent sleep deprivation or non‐sleep deprivation experiments. Offspring male mice were used as experimental subjects for drug treatment and behavioral and molecular experiments (see Figure 1). All animals were housed in a standard environment with a 12‐h light–dark cycle, a temperature of 21–23°C, humidity of 50%–60%, and freely available food and water.

### 2.2 Sleep deprivation

The sleep deprivation experiments have been previously described (Zhang et al., 2023a). The sleep deprivation device can put four mice on the moving belt at a time, and the dimensions of this apparatus are 630 long, 565 wide, and 310 mm high (see Figure 1). We placed the mice in the apparatus for 2 h each day for 3 consecutive days to acclimate the environment before performing the sleep deprivation test. Pregnant female mice were placed in a sleep deprivation apparatus for 6 h per day (12:00–18:00 h) for 7 days during late gestation (GD15–21). The sleep deprivation apparatus kept the mice awake by means of a continuously moving belt at a speed of 0.5 m/min. Meanwhile, to avoid confusion from environmental factors, other pregnant females in the control group were placed in a sleep deprivation machine whose belt did not move. The mice had free access to food and water during the sleep deprivation period.

### 2.3 Melatonin treatment

After the male offspring mice had been reared to 2 months old, they were divided into four groups: Control + saline, Control + Mel, MSD + saline, and MSD + Mel. There were eight mice in each group. Mice in the Control + Mel and MSD + Mel groups were given 20 mg/kg Mel by intraperitoneal injection per day (Sigma‐Aldrich, M5250, powder, purity ≥98%) for 28 days. The choice of Mel concentration of 20 mg/kg was based on previous studies (Lin et al., 2020; Veschsanit et al., 2021). The Control + saline and the MSD + saline groups were administered the same volume of saline by intraperitoneal injection.

### 2.4 Morris water maze test

The water maze experiment was used to assess the spatial learning and memory abilities of mice as in previous studies. During the learning phase, mice were placed in the water from different quadrants facing the barrel wall four times a day for 5 days. Regardless of whether the mice could or could not find the underwater target platform within 60 s for each training session, they were allowed to rest on the platform for 30 s. Mice that did not find the target platform within 60 s were guided to the target platform by the experimenter. During the spatial exploration phase, the target platform was removed 2 h after the last training session, and mice were placed in the pool from the opposite quadrant of the target targetivity and allowed to explore freely for 60 s. Any‐Maze software (Stoelting) recorded and analyzed escape latency, swimming velocity, and distance during the learning period, as well as the percentages of time and distance from the target quadrant during the memory period.

### 2.5 Enzyme‐linked immunosorbent assay

Mice were anesthetized with 2% pentobarbital sodium before being euthanized. The brain was then rapidly removed, and hippocampal tissue was isolated and removed on ice and stored in a −80°C refrigerator for further molecular experiments. The hippocampal tissue was homogenized using a homogenizer and centrifuged for about 20 min (2000–3000 rpm); subsequently, the supernatant was carefully collected. Concentrations of IL‐1β, IL‐6, and TNF‐α were determined according to the instructions of the Enzyme‐linked Immunoassay Kit for Mice from Wuhan ColorfulGene Biological Technology Co., ltd (JYM0531Mo, JYM0012Mo, and JYM0218Mo). Specifically, the experimental steps include dilution of standards, leaving a well empty as blank control in the microelisa stripplate, adding 50 µL HRP‐conjugate reagent to each well except the blank control well, incubation, dilution, washing, coloring, and termination. And in addition, I finally read absorbance O.D. at 450 nm using a microtiter plate reader.

### 2.6 Reverse transcription‐polymerase chain reaction (RT‐PCR)

Total RNA was extracted from the ground hippocampal tissue using TRIzol lysis (Life Technologies, 15596018). The purity of total RNA was measured using an ultramicro photometer. Based on our previous study (Zhang et al., 2023b), cDNA was synthesized using a PrimeScript RT reagent Kit with gDNA Eraser (Takara, RR047A). Reverse transcription‐polymerase chain reaction was performed using a 2 × SYBR Green mixture of 5 µL, forward primer of 1 µL, reverse primer of 1 µL, cDNA of 1 µL, and RNase free water of 2 µL. The relative quantification of RNA expression was performed by the 2^−ΔΔ^ *^Ct^* method. The primer sequences used were as follows (Table 1).

**TABLE 1:** Reverse transcription‐polymerase chain reaction (RT‐PCR) primers’ sequence details.

Gene	Amplicon size (bp)	Forward primer (5′ → 3′)	Reverse primer (5′ → 3′)
β‐actin	120	AGTGTGACGTTGACATCCGT	TGCTAGGAGCCAGAGCAGTA
BDNF	94	TTACTCTCCTGGGTTCCTGA	ACGTCCACTTCTGTTTCCTT
TrkB	104	TCTGGAGGGTGCTATGCTAT	GGGGCAGAAACTCCAGAAAA
PSD95	72	CCCAGGATATGTGAACGGAA	CCTGAGTTACCCCTTTCCAA
SYN	124	GCCTACCTTCTCCACCCTTT	GCACTACCAACGTCACAGAC

Abbreviations: BDNF, brain‐derived neurotrophic factor; PSD‐95, postsynaptic density protein‐95; SYN, synaptophysin.

### 2.7 Western blotting

The RIPA cell lysis solution (Beyotime, P0013B) was added to the hippocampal tissue for lysis. It was then centrifuged at 12,000 × *g* for 15 min, after which the supernatant was carefully collected. Protein samples were loaded into SDS–PAGE gel spiked wells. Following constant pressure 80 v electrophoresis for 1 h, samples were transferred to a PVDF membrane (Millipore, IPVH00010), specific transfer times were 60 min for PSD‐95, 35 min for SYN, 20 min for BDNF, and 65 min for TrkB. After the film transfer, western blocking solution (5% skim milk powder) was added and blocked for 2 h at room temperature. Then, the membranes were incubated with diluted rabbit antibodies against PSD‐95 (1:2000, Abcam, ab238135), SYN (1:1000, Bioss, bs‐8845R), BDNF (1:1000, Abcam, ab108319), and TrkB (1:5000, Abcam, ab187041) overnight at 4°C. Membranes were then incubated for 1.2 h at room temperature with a horseradish peroxidase‐conjugated goat anti‐rabbit immunoglobulin G (1:20000, Zsbio, ZB‐2301). Proteins were detected using an ECL ultrasensitive luminescence kit (Thermo, 340958) according to the manufacturer's instructions. ImageJ software was used for bands analysis (Media Cybernetics).

### 2.8 Statistical analysis

Statistical analysis of all data was performed with Prism 8 (GraphPad Software, Inc.). Results are expressed as means ± standard error of the mean. The intergroup variation was measured by one way analysis of variance (ANOVA) for all tests followed by post hoc multiple comparison with Tukey's test, except in the escape latency, swimming velocity, and distance of Morris water maze test, where two way ANOVA was applied. The correlations between behavioral and molecular indicators were analyzed using Pearson's correlation test. A *p*‐value <.05 was considered significant.

## 3 RESULTS

### 3.1 Melatonin alleviated MSD‐induced cognitive deficits in murine male offspring

**FIGURE 2:**
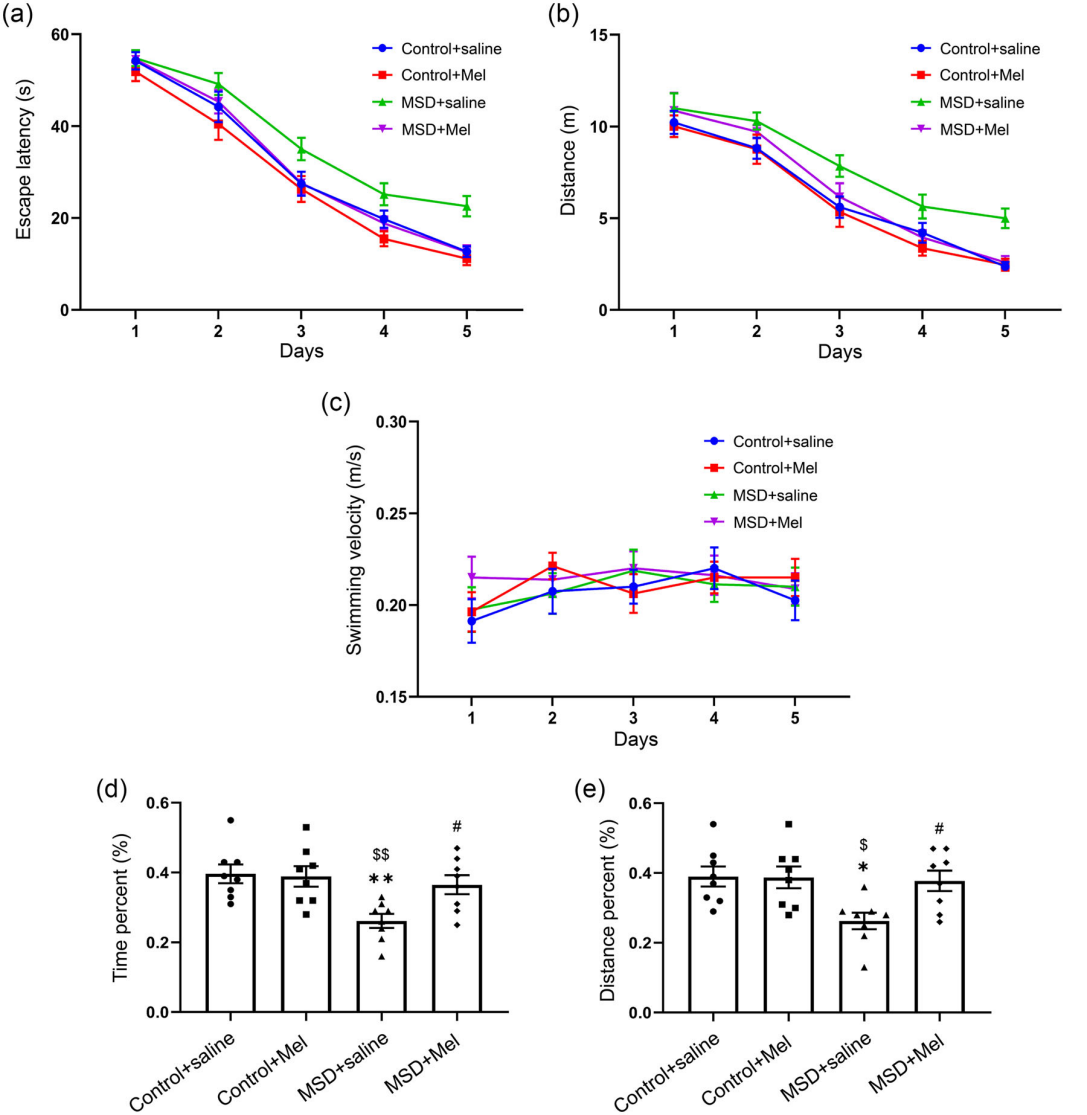
Effect of melatonin on maternal sleep deprivation‐induced learning and memory impairment in the Morris water maze test. In the learning phase, the escape latency (a), distance (b), and swimming velocity (c) are shown. In the memory phase, the percentages of time (d) and distance (e) in the target quadrant are shown. ^*^ *p* < .05, ^**^ *p* < .01 versus Control + saline group; ^$^ *p* < .05, ^$$^ *p* < .01 versus Control + Mel group; ^#^ *p* < .05 versus MSD + saline group. *N* = 8 per group.

In the learning phase, there were no significant differences in the swimming velocity among the four groups as shown in Figure 2. However, the escape latency and swimming distances were longer in the MSD + saline group than the Control + saline and Control + Mel groups (*p* < .05). Moreover, the escape latency and swimming distance were significantly shorter in the MSD + Mel group than the MSD + saline group (*p* < .05).

In the memory period, significant changes in the percentages of time and distance swam in the target quadrant were found between the four groups using one‐way ANOVA (time percent: *F*_(3,28)_ = 5.77, *p* < .01; distance percent: *F*_(3,28)_ = 4.69, *p* < .01; Figure 2d,e). The percentages of time and distance swam in the target quadrant were shortened when mice were exposed to MSD compared with those from the Control + saline and Control + Mel groups (*p* < .05). However, compared to MSD exposure, Mel administration increased the percentages of time and distance swam in the target quadrant (*p *< .05).

### 3.2 Melatonin suppressed the MSD‐induced increase in inflammatory factor levels

**FIGURE 3:**
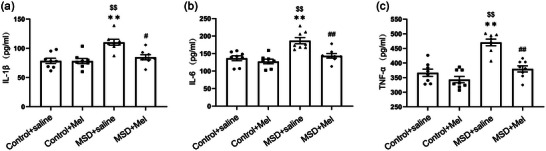
Effects of maternal sleep deprivation and melatonin (Mel) on expression levels of interleukin (IL)‐1β, IL‐6, and tumor necrosis factor‐α (TNF‐α) in the hippocampus. The expression levels of hippocampal IL‐1β1 (a), IL‐6 (b), and TNF‐α (c) are shown. ^**^ *p* < .01 versus Control + saline group; ^$$^ *p* < .01 versus Control + Mel group; ^#^ *p* < .05, ^##^ *p* < .01 versus MSD + saline group. *N* = 8 per group.

The results showed that Mel treatments had a significant effect on the levels of IL‐1β, IL‐6, and TNF‐α in the hippocampus (IL‐1β: *F*_(3,28)_ = 10.85, *p* < .01; IL‐6: *F*_(3,28)_ = 13.15, *p* < .01; TNF‐α: *F*_(3,28_) = 24.37, *p* < .01; Figure 3a–c). The IL‐1β, IL‐6, and TNF‐α levels were significantly increased in the MSD + saline group as compared to Control + saline and Control + Mel groups (*p *< .05). Nevertheless, the administration of Mel significantly mitigated this increase of IL‐1β, IL‐6, and TNF‐α levels when male offspring mice were exposed to MSD (*p* < .05).

### 3.3 Effect of melatonin on MSD‐induced changes in mRNA levels of BDNF, TrkB, PSD‐95, and SYN

**FIGURE 4:**
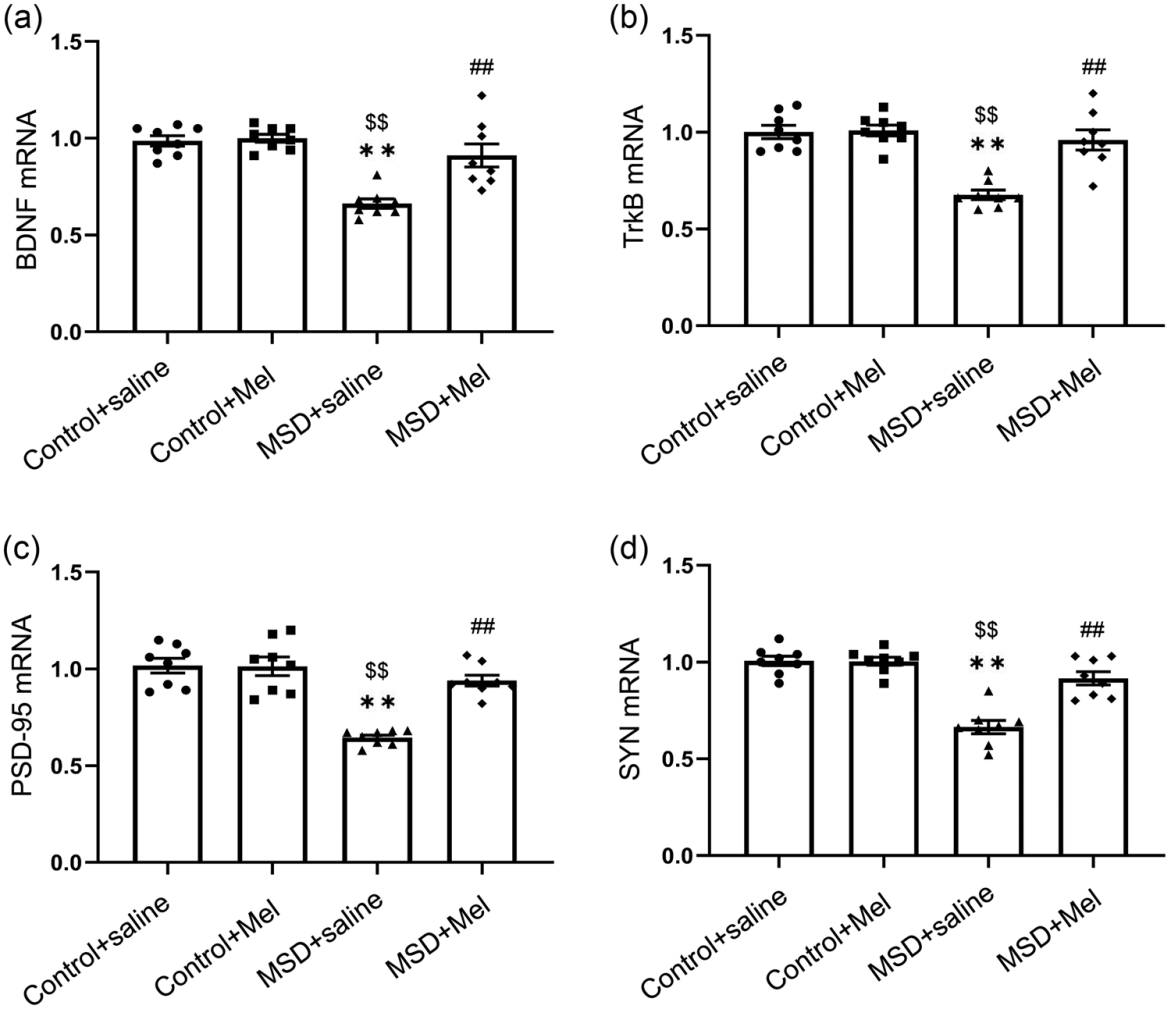
Effects of maternal sleep deprivation and melatonin on brain‐derived neurotrophic factor (BDNF), TrkB, postsynaptic density protein‐95 (PSD‐95), and synaptophysin (SYN) mRNA levels in the hippocampus. The mRNA levels of hippocampal BDNF (a), TrkB (b), PSD‐95 (c), and TSYN (d) are shown. ^**^ *p* < .01 versus Control + saline group; ^$$^ *p* < .01 versus Control + Mel group; ^##^ *p* < .01 versus MSD + saline group. *N* = 8 per group.

As shown in Figure 4, BDNF, TrkB, PSD‐95, and SYN mRNA levels in the hippocampus were significantly different among the four groups (BDNF: *F*_(3,28)_ = 18.40, *p* < .01; TrkB: *F*_(3,28)_ = 19.31, *p *< .01; PSD‐95: *F*_(3,28)_ = 26.10, *p *< .01; SYN: *F*_(3,28)_ = 30.29, *p* < .01; Figure 4a–d). Hippocampal BDNF, TrkB, PSD‐95, and SYN mRNA levels markedly decreased in MSD + saline mice as compared to Control + saline mice (*p* < .05). Treatment with Mel produced a statistically significant increase in the mRNA levels of BDNF, TrkB, PSD‐95, and SYN as compared to those in the MSD + saline group (*p* < .05).

### 3.4 Effect of melatonin on MSD‐induced changes in the protein levels of BDNF, TrkB, PSD‐95, and SYN

**FIGURE 5:**
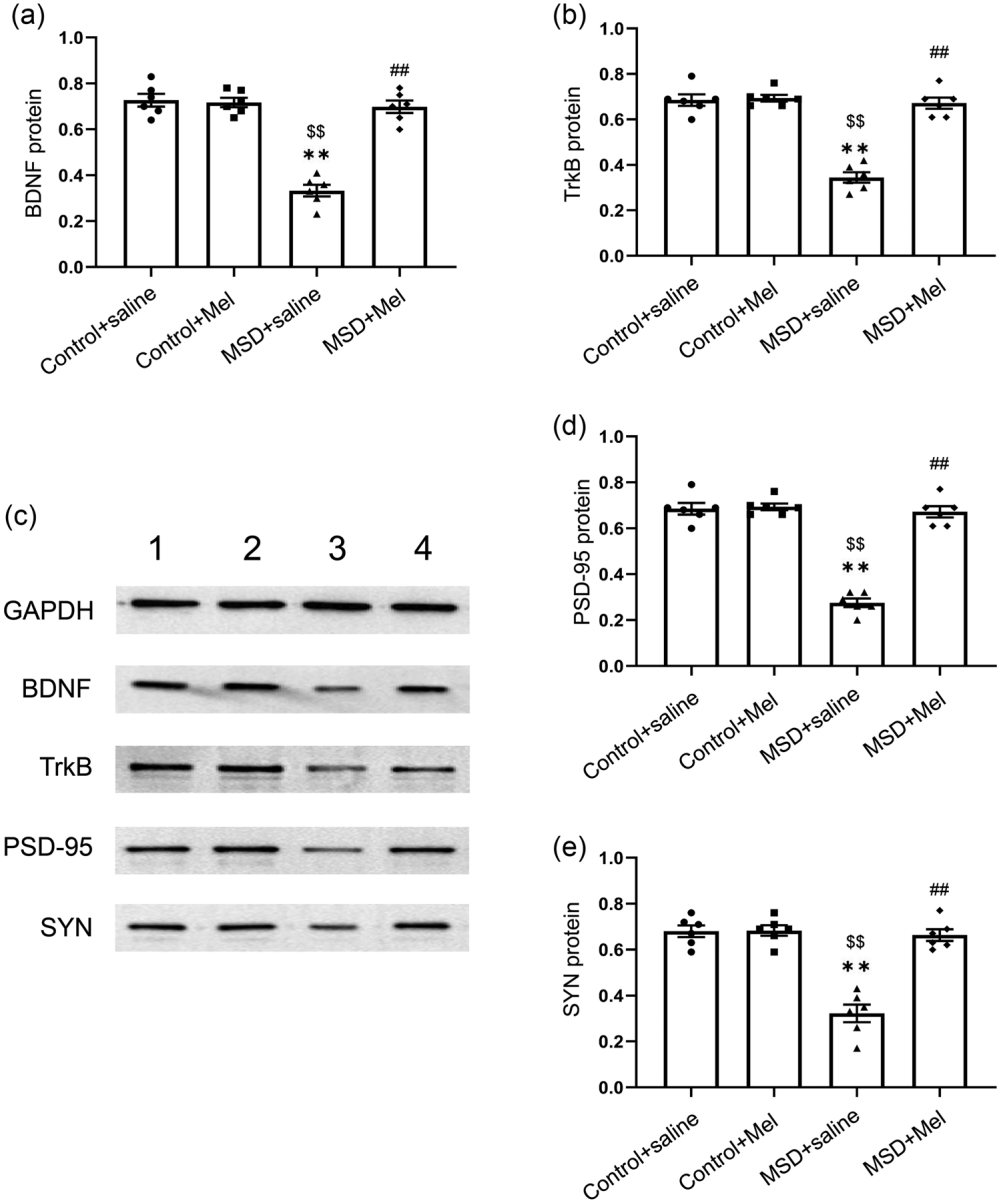
Effects of maternal sleep deprivation and melatonin on brain‐derived neurotrophic factor (BDNF), TrkB, postsynaptic density protein‐95 (PSD‐95), and synaptophysin (SYN) protein levels in the hippocampus. The protein levels of hippocampal BDNF (a), TrkB (b), PSD‐95 (d), and TSYN (e) are shown. (c) Western blot: band 1: Control + saline group; band 2: Control + Mel group; band 3: MSD + saline group; band 4: MSD + Mel group ^**^ *p* < .01 versus Control + saline group; ^$$^ *p* < .01 versus Control + Mel group; ^##^ *p* < .01 versus MSD + saline group. *N* = 6 per group.

One‐way ANOVA showed that protein levels of BDNF, TrkB, PSD‐95, and SYN in the hippocampus differed significantly among the four groups (BDNF: *F*_(3,20)_ = 56.43, *p* < .01; TrkB: *F*_(3,20)_ = 22.83, *p *< .01; PSD‐95: *F*_(3,20)_ = 57.86, *p* < .01; SYN: *F*_(3,20)_ = 38.14, *p* < .01; Figure 5a–e). MSD significantly reduced BDNF, TrkB, PSD‐95, and SYN protein expression levels compared with mice in Control + saline and Control + Mel groups (*p* < .05). However, BDNF, TrkB, PSD‐95, and SYN protein expression levels were reversed in the Mel treatment group compared with the stress group (*p* < .05).

### 3.5 Correlations between cognitive performance and the levels of synaptic plasticity‐associated proteins and proinflammatory cytokines

#### 3.5.1 Correlation with the levels of IL‐1β, IL‐6, and TNF‐α

Results showed that the levels of IL‐1β, IL‐6, and TNF‐α in all groups were positively correlated with the escape latency and distance swam in the learning phase (*p* < .05); they were also negatively correlated with the percentages of time and distance swam in the memory phase in the Morris water maze test (*p* < .05), see Table 2.

**TABLE 2:** Correlation between the performance in the cognition‐related tasks and hippocampal levels of interleukin (IL)‐1β, IL‐6, and tumor necrosis factor‐α (TNF‐α) [*r*(*p*)].

Tasks	Indexes	Groups	Proinflammatory cytokines		
			IL‐1β	IL‐6	TNF‐α
Morris water maze test	Escape latency	Control + saline	0.753 (0.031)*	0.630 (0.094)	0.810 (0.015)*
		Control + Mel	0.936 (0.001)**	0.837 (0.010)**	0.857 (0.007)**
		MSD + saline	0.792 (0.019)*	0.762 (0.028)*	0.964 (0.000)**
		MSD + Mel	0.930 (0.001)**	0.958 (0.000)**	0.819 (0.013)*
	Swimming distance	Control + saline	0.592 (0.122)	0.480 (0.229)	0.661 (0.074)
		Control + Mel	0.947 (0.000)**	0.843 (0.009)**	0.839 (0.009)**
		MSD + saline	0.882 (0.004)**	0.970 (0.000)**	0.839 (0.009)**
		MSD + Mel	0.861 (0.006)**	0.829 (0.011)*	0.855 (0.007)**
	Percentage of time swam	Control + saline	0.579 (0.133)	0.517 (0.189)	0.475 (0.234)
		Control + Mel	−0.863 (0.006)**	−0.848 (0.008)**	−0.799 (0.017)*
		MSD + saline	−0.895 (0.003)**	−0.968 (0.000)**	−0.831 (0.011)*
		MSD + Mel	−0.921 (0.001)**	−0.896 (0.003)**	−0.888 (0.003)**
	Percentage of distance swam	Control + saline	0.477 (0.232)	0.432 (0.285)	0.384 (0.348)
		Control + Mel	−0.832 (0.010)*	−0.784 (0.021)*	−0.772 (0.025)*
		MSD + saline	−0.963 (0.000)**	−0.919 (0.001)**	−0.823 (0.012)*
		MSD + Mel	−0.871 (0.005)**	−0.842 (0.009)**	−0.857 (0.007)**

Abbreviations: Mel, melatonin; MSD, maternal sleep deprivation. *Denotes significant correlation (*P < 0.05; **P < 0.01)

#### 3.5.2 Correlation with the mRNA levels of BDNF, TrkB, PSD‐95, and SYN

The results showed that the mRNA levels of BDNF, TrkB, PSD‐95, and SYN in all groups were negatively correlated with the escape latency and distance swam in the learning phase (*p* < .05) and positively correlated with the percentages of time and distance swam in the memory phase in the Morris water maze test (*p* < .05), see Table 3.

**TABLE 3:** Correlation between the performance in the cognition‐related tasks and hippocampal mRNA levels of brain‐derived neurotrophic factor (BDNF), TrkB, postsynaptic density protein‐95 (PSD‐95), and synaptophysin (SYN) [*r*(*p*)].

Tasks	Indexes	Groups	mRNA			
			BDNF	TrkB	PSD‐95	SYN
Morris water maze test	Escape latency	Control + saline	−0.397 (0.330)	−0.725 (0.042)*	−0.541 (0.166)	−0.776 (0.024)*
		Control + Mel	−0.884 (0.004)**	−0.921 (0.001)**	−0.762 (0.028)*	−0.967 (0.000)**
		MSD + saline	−0.883 (0.004)**	−0.737 (0.037)*	−0.720 (0.044)*	−0.902 (0.002)**
		MSD + Mel	−0.791 (0.019)*	−0.908 (0.002)**	−0.871 (0.005)**	−0.834 (0.010)*
	Swimming distance	Control + saline	−0.312 (0.452)	−0.691 (0.058)	−0.570 (0.140)	−0.556 (0.153)
		Control + Mel	−0.918 (0.001)**	−0.948 (0.000)**	−0.812 (0.014)*	−0.978 (0.000)**
		MSD + saline	−0.840 (0.009)**	−0.760 (0.028)*	−0.967 (0.000)**	−0.822 (0.012)*
		MSD + Mel	−0.885 (0.003)**	−0.867 (0.005)**	−0.842 (0.009)**	−0.990 (0.000)**
	Percentage of time swam	Control + saline	−0.715 (0.046)*	−0.496 (0.212)	−0.646 (0.084)	−0.498 (0.209)
		Control + Mel	0.882 (0.004)**	0.899 (0.002)**	0.760 (0.029)*	0.897 (0.003)**
		MSD + Saline	0.845 (0.008)**	0.798 (0.018)*	0.948 (0.000)**	0.866 (0.005)**
		MSD + Mel	0.850 (0.007)**	0.920 (0.001)**	0.830 (0.011)*	0.958 (0.000)**
	Percentage of distance swam	Control + saline	−0.677 (0.065)	−0.376 (0.359)	−0.553 (0.155)	−0.407 (0.317)
		Control + Mel	0.874 (0.005)**	0.876 (0.004)**	0.696 (0.055)	0.879 (0.004)**
		MSD + saline	0.867 (0.005)**	0.813 (0.014)*	0.899 (0.002)**	0.913 (0.002)**
		MSD + Mel	0.786 (0.021)*	0.871 (0.005)**	0.764 (0.027)*	0.959 (0.000)**

Abbreviations: Mel, melatonin; MSD, maternal sleep deprivation.*Denotes significant correlation (*P < 0.05; **P < 0.01).

#### 3.5.3 Correlation with protein levels of BDNF, TrkB, PSD‐95, and SYN

Linear correlation analyses showed that a significant negative correlation occurred between escape latency and BDNF, TrkB, PSD‐95, and SYN protein levels in all groups (*p* < .05), as well as between swimming distance of the learning period and those protein levels (*p* < .05). Moreover, the percentages of time and distance in the target quadrant were positively correlated with BDNF, TrkB, PSD‐95, and SYN protein levels in all groups (*p* < .05), see Table 4.

**TABLE 4:** Correlation between the performance in the cognition‐related tasks and hippocampal protein levels of brain‐derived neurotrophic factor (BDNF), TrkB, postsynaptic density protein‐95 (PSD‐95), and synaptophysin (SYN) [*r*(*p*)].

Tasks	Indexes	Groups	Synaptic proteins			
			BDNF	TrkB	PSD‐95	SYN
Morris water maze test	Escape latency	Control + saline	−0.761 (0.079)	−0.704 (0.118)	−0.721 (0.106)	−0.714 (0.111)
		Control + Mel	−0.976 (0.001)**	−0.884 (0.019)*	−0.888 (0.018)*	−0.982 (0.000)**
		MSD + saline	−0.824 (0.044)*	−0.896 (0.016)*	−0.855 (0.030)*	−0.804 (0.054)
		MSD + Mel	−0.975 (0.001)**	−0.807 (0.052)	−0.859 (0.028)*	−0.867 (0.025)*
	Swimming distance	Control + saline	−0.650 (0.162)	−0.480 (0.336)	−0.433 (0.391)	−0.563 (0.245)
		Control + Mel	−0.954 (0.003)**	−0.936 (0.006)**	−0.947 (0.004)**	−0.974 (0.001)**
		MSD + saline	−0.975 (0.001)**	−0.976 (0.001)**	−0.990 (0.000)**	−0.970 (0.001)**
		MSD + Mel	−0.925 (0.008)**	−0.844 (0.035)*	−0.913 (0.011)*	−0.957 (0.003)**
	Percentage of time swam	Control + saline	−0.452 (0.368)	−0.392 (0.442)	−0.392 (0.442)	−0.548 (0.260)
		Control + Mel	0.931 (0.007)**	0.948 (0.004)**	0.951 (0.003)**	0.892 (0.017)*
		MSD + saline	0.955 (0.003)**	0.907 (0.013)*	0.956 (0.003)**	0.973 (0.001)**
		MSD + Mel	0.960 (0.002)**	0.894 (0.016)*	0.954 (0.003)**	0.980 (0.001)**
	Percentage of distance swam	Control + saline	−0.326 (0.528)	−0.292 (0.575)	−0.312 (0.547)	−0.449 (0.372)
		Control + Mel	0.944 (0.005)**	0.909 (0.012)*	0.940 (0.005)**	0.869 (0.025)*
		MSD + saline	0.958 (0.003)**	0.872 (0.024)*	0.922 (0.009)**	0.965 (0.002)**
		MSD + Mel	0.962 (0.002)**	0.855 (0.030)*	0.949 (0.004)**	0.959 (0.002)**

Abbreviations: Mel, melatonin; MSD, maternal sleep deprivation.*Denotes significant correlation (*P < 0.05; **P < 0.01)

## 4 DISCUSSION

Results of the current study showed the potential therapeutic role of Mel to mitigate MSD‐induced spatial learning and memory impairment and changes in inflammation and synaptic function in the hippocampi of murine offspring. MSD‐induced offspring mice showed learning and memory impairment as observed in the Morris water maze test, which is associated with changes in markers of inflammation and synaptic function. Administration of Mel reversed MSD‐induced cognitive impairments partly through improving inflammation and synaptic dysfunction. Collectively, our study highlights the beneficial effects of Mel as a potential agent in murine MSD‐induced offspring.

### 4.1 Melatonin improved MSD‐induced learning and memory impairment

Intrauterine fetal development includes an essential period for brain development which is susceptible to stressful events. These can lead to neuropsychiatric disorders in offspring during adulthood/aging, which can be the developmental origins of health and disease. It is reported that chronic restraint stress from GD 12 to 18 increases anxiety‐ and depression‐like behaviors in rat offspring (Lei et al., 2020). Our previous studies document that prenatal inflammation exposure significantly aggravates aging‐related learning and memory dysfunction (Ni et al., 2022; Zhuang et al., 2021). In the present study, the MSD‐induced mice displayed increased escape latency and distance during the learning phase and a decreased time and distance percent in the target quadrant during the memory phase, suggesting cognitive impairment induced by MSD. The results are in accordance with a previous study demonstrating that the offspring rats exposed to sleep deprivation on GD 18 for 72 h showed impaired hippocampal‐dependent learning and memory in the Morris water maze test (Zhao et al., 2014). Growing evidence suggests that a low level of Mel in the brain and plasma is closely related to cognitive dysfunction. Specifically, exogenous Mel supplementation has reversed Alzheimer's disease‐ and vascular dementia‐associated cognitive deficits (Thangwong et al., 2022). Consistently, our results showed that treatment with Mel prevented MSD‐induced learning and memory impairment as demonstrated by the decrease in the escape latency and distance and increase in the percent time and distance in the MSD + Mel group compared to MSD group.

Additionally, there were no significant differences in swimming speed among the four groups during the Morris water maze test. This indicates that the beneficial effects of Mel on MSD‐induced cognitive impairment were not related to alterations in motor activity.

### 4.2 Melatonin improved MSD‐induced inflammation in the hippocampus

Inflammatory cells in the brain, predominantly microglial, monitor the physiological environment of the brain. When stimulated by external pathological factors, the activated microglial cells lead to excessive release of proinflammatory cytokines including IL‐1β, IL‐6, and TNF‐α through downstream signaling pathways, resulting in neuroinflammation (de Sousa et al., 2023; Ni et al., 2023). This neuroinflammation may affect cell proliferation, differentiation, neuronal activity, and neuronal network and is closely associated with cognitive function (Palmisano et al., 2022; Yildirim et al., 2023). The prenatal stress‐induced proinflammatory mediators may activate microglia in the offspring's brain through maternal–placental–fetal inflammatory pathways, accompanied by an increase in proinflammatory cytokines, which can lead to adult/aging susceptibility to affective disorders, and learning and memory dysfunction (Entringer et al., 2010; Perry et al., 2003; Williamson et al., 2011). A previous study shows that a MSD induced microglial shift to a proinflammatory morphology, increases the expression of pro‐inflammatory cytokines IL‐1β, IL‐6, and TNF‐α, and decreases the expression of anti‐inflammatory cytokine IL‐10 in the hippocampus of rat offspring (Zhao et al., 2014). These phenomena contribute to the cognitive impairment induced by MSD. In full agreement with these previous studies, our results demonstrated that MSD significantly increased IL‐1β, IL‐6, and TNF‐α levels in the hippocampus of offspring mice, which partly explained their poor performance in the Morris water maze test.

Emerging evidence suggests that Mel can enhance cognition by improving inflammation; in this study, Mel shifted microglia into an anti‐inflammatory phenotype via a sirtuin1‐dependent manner to attenuate BDE‐209‐induced cognitive impairment (Wu et al., 2023). Furthermore, Mel can ameliorate methamphetamine‐induced cognitive impairment, as the anti‐inflammatory agent minocycline, by decreasing elevated levels of proinflammatory cytokines in the blood serum and hippocampus (Lwin et al., 2021). Our recent report shows that a long‐term enriched environment could overcome MSD‐induced cognitive dysfunction in murine offspring through improving inflammatory responses in the hippocampus (Zhang et al., 2023b). In the current study, Mel significantly decreased the expression levels of IL‐1β, IL‐6, and TNF‐α in the MSD + Mel group compared to the MSD + saline group and subsequently ameliorated MSD‐induced spatial learning and memory impairment.

### 4.3 Melatonin improved MSD‐induced synaptic dysfunction in the hippocampus

Hippocampal synaptic plasticity is closely related to cognitive function and is heavily reliant on the BDNF/TrkB signaling pathway, which regulates dendritic growth, neurogenesis, synaptic maturation, synaptic transmission, and synaptic protein synthesis (Liu et al., 2023; Wang et al., 2023). It is well known that prenatal stress interferes with synaptic pruning and synaptic plasticity in the hippocampus through environmental factors and brain counteractions via epigenetic mechanisms (Dong et al., 2018; Li et al., 2021). The BDNF/TrkB signaling pathway has been downregulated in rats exposed to maternal restraint stress accompanied by spatial learning and memory and working memory deficits (Zheng et al., 2020). Similarly, MSD also downregulated the BDNF/TrkB signaling pathway, as shown by the decreased mRNA and protein levels of BDNF and TrkB in MSD + saline offspring, compared to the Control + saline group. The presynaptic protein (SYN) and postsynaptic protein (PSD‐95) are activated by the BDNF/TrkB signaling pathway and play an important role in synaptic plasticity (Shen et al., 2019). In addition, decreased levels of PSD‐95 and SYN in the hippocampus are associated with cognitive impairment induced by acute sleep deprivation (Farajdokht et al., 2021). Our results showed that the mRNA and protein levels of PSD‐95 and SYN were decreased in MSD offspring, which contributed to the synaptic dysfunction caused by MSD.

Several studies report that Mel regulates dendritic arborization, synaptic plasticity, and synaptic proteins in the hippocampus (El‐Sherif et al., 2003; Pascual & Bustamante, 2011; Ramírez‐Rodríguez et al., 2018). Mel also improved learning and memory disturbances in a diabetic model through preventing dendritic spine loss (Albazal et al., 2021). Alzheimer's disease is a neurodegenerative disease in which the associated cognitive decline is accompanied by an alteration in synaptic proteins in the hippocampus. The cognitive deficits and reduction in synaptosomal‐associated protein 25 and SYN induced by Aβ1‐42, which is used to create a model of Alzheimer's disease, were completely abolished by Mel treatment (Zhang et al., 2016). In the current study, our results further revealed that Mel reversed the MSD‐induced decrease in SYN and PSD‐95 by modulating the BDNF/TrkB signaling pathway. These results imply that Mel has a beneficial effect on synaptic dysfunction induced by MSD.

### 4.4 Correlation between cognition‐associated performance and markers of inflammation and synaptic function

There is a correlation between cognitive deficits and inflammation and synaptic dysfunction. Furthermore, elevated inflammatory cytokines in the hippocampus are correlated with cognitive impairment associated with neurodegenerative diseases (Li et al., 2023; N Danappanvar et al., 2023). The present study found that the levels of IL‐1β, IL‐6, and TNF‐α in the hippocampus were correlated with indicators of the Morris water maze test in the MSD model. The correlation between the aging‐associated cognitive decline and changes in synaptic proteins, such as PSD‐95, synaptotagmin‐1, and activity‐regulated cytoskeleton‐associated protein, have been reported (Ni et al., 2022; Zhang, Zeng, et al., 2022). Our results showed that the mRNA and protein levels of BDNF, TrkB, PSD‐95, and SYN were correlated with performance in the Morris water maze test. Collectively, it is reasonable to assume that the beneficial effect of Mel on cognitive impairment induced by MSD is related to improvements in hippocampal inflammation and synaptic dysfunction.

There are some limitations in our study. First, MSD has a sex effect on offspring's neurobehavior and the therapeutic effects of Mel were only determined in male offspring. Second, we did not examine the effects of Mel on markers of inflammation and synaptic function in other brain regions associated with cognition. Third, we did not evaluate the morphological and histological changes in the hippocampus. Finally, we did not evaluate the markers of oxidative stress and apoptosis to further validate the neuroprotective actions of Mel.

## 5 CONCLUSION

In conclusion, the current findings support previous reports that MSD leads to impairments in spatial learning and memory in offspring, which are associated with inflammation and synaptic dysfunction in the hippocampus. Mel mitigates MSD‐induced cognitive deficits through improving inflammation and synaptic dysfunction. Therefore, these results suggest that the supplementation of exogenous Mel could be a new pharmacological approach to the treatment of cognitive impairment caused by MSD.

## AUTHOR CONTRIBUTIONS

**Yue‐Ming Zhang**: Conceptualization; investigation; methodology; validation; software; formal analysis; data curation; supervision; writing—original draft; writing—review and editing. **Ru‐Meng Wei**: Writing—original draft; writing—review and editing; conceptualization; methodology; software; formal analysis; project administration; data curation; supervision. **Zong‐Yin Li**: Writing—original draft; visualization; validation; methodology; formal analysis; software. **Xue‐Yan Li**: Writing—review and editing; project administration; conceptualization; investigation. **Kai‐Xuan Zhang**: Conceptualization; investigation; validation; formal analysis; writing—review and editing. **Yi‐Jun Ge**: Conceptualization; investigation; formal analysis; validation; writing—review and editing. **Xiao‐Yi Kong**: Writing—review and editing; methodology; conceptualization; investigation; funding acquisition; formal analysis. **Xue‐Chun Liu**: Methodology; writing—original draft; writing—review and editing; formal analysis; software; conceptualization; investigation; data curation; supervision; funding acquisition. **Gui‐Hai Chen**: Funding acquisition; conceptualization; methodology; validation; project administration; formal analysis; resources; software; supervision; visualization; writing—review and editing; writing—original draft; investigation; data curation.

## CONFLICT OF INTEREST STATEMENT

The authors declare that the research was conducted in the absence of any commercial or financial relationships that could be construed as a potential conflicts of interest.

## FUNDING INFORMATION

National Natural Science Foundation of China, Grant Number: 81671316; 2022 Key Research and Development Plan of Anhui Province, Grant Number: 2022e07020029; College of the Natural Science Foundation of Anhui Province, Grant Number: 2022AH050759

### PEER REVIEW

The peer review history for this article is available at https://publons.com/publon/10.1002/brb3.3515.

## Data Availability

The raw data supporting the conclusions of this article will be made available by the authors without undue reservation.
